# A Camelid-Derived STAT-Specific Nanobody Inhibits Neuroinflammation and Ameliorates Experimental Autoimmune Encephalomyelitis (EAE)

**DOI:** 10.3390/cells13121042

**Published:** 2024-06-16

**Authors:** Evaristus C. Mbanefo, Allison Seifert, Manoj Kumar Yadav, Cheng-Rong Yu, Vijayaraj Nagarajan, Ashutosh Parihar, Sunanda Singh, Charles E. Egwuagu

**Affiliations:** 1 Molecular Immunology Section, Laboratory of Immunology, National Eye Institute (NEI), National Institutes of Health (NIH), Bethesda, MD 20892, USA; 2 Singh Biotechnology, 1547 Fox Grape Loop, Lutz, FL 33558, USA

**Keywords:** nanobody, SBT-100, encephalomyelitis, EAE, autoimmunity, STAT1, STAT3, SH2 domain, Th17, Th1

## Abstract

Proinflammatory T-lymphocytes recruited into the brain and spinal cord mediate multiple sclerosis (MS) and currently there is no cure for MS. IFN-γ-producing Th1 cells induce ascending paralysis in the spinal cord while IL-17-producing Th17 cells mediate cerebellar ataxia. STAT1 and STAT3 are required for Th1 and Th17 development, respectively, and the simultaneous targeting of STAT1 and STAT3 pathways is therefore a potential therapeutic strategy for suppressing disease in the spinal cord and brain. However, the pharmacological targeting of STAT1 and STAT3 presents significant challenges because of their intracellular localization. We have developed a STAT-specific single-domain nanobody (SBT-100) derived from camelids that targets conserved residues in Src homolog 2 (SH2) domains of STAT1 and STAT3. This study investigated whether SBT-100 could suppress experimental autoimmune encephalomyelitis (EAE), a mouse model of MS. We show that SBT-100 ameliorates encephalomyelitis through suppressing the expansion of Th17 and Th1 cells in the brain and spinal cord. Adoptive transfer experiments revealed that lymphocytes from SBT-100-treated EAE mice have reduced capacity to induce EAE, indicating that the immunosuppressive effects derived from the direct suppression of encephalitogenic T-cells. The small size of SBT-100 makes this STAT-specific nanobody a promising immunotherapy for CNS autoimmune diseases, including multiple sclerosis.

## 1. Introduction

The Janus kinase/signal transducer and activator of transcription factor (JAK/STAT) is an evolutionarily conserved signaling pathway that transduces extracellular signals to the nucleus, and regulates genes that influence the behavior of cells in response to its extracellular environmental stimuli [1]. There are four Janus kinases (JAK1, JAK2, JAK3, Tyk2) and seven members of the STAT family (STAT1, STAT2, STAT3, STAT4, STAT5a, STAT5b, and STAT6). STAT1 and STAT3 play critical roles in the development of the proinflammatory lymphocyte subsets, Th1 and Th17, and they also regulate the initiation, duration and intensity of proinflammatory immune responses [2,3]. The binding of a cytokine to its cognate receptor on a lymphocyte results in transphosphorylation and the activation of receptor-associated JAKs, which in turn recruits specific STATs and phosphorylate, a tyrosine residue within the SH2 domain of the STAT protein. The tyrosine-phosphorylated STATs form homo- or hetero-dimers via reciprocal phosphotyrosine-SH2 interactions. The dimers translocate into the nucleus where they bind specific DNA sequences or GAS (Gamma-interferon activation site) and activate or repress gene transcription [2,3]. Latent unphosphorylated STAT (U-STAT) proteins in the cytoplasm also form stable dimers and associate with members of the importin family proteins that transport them into the nucleus where they activate or repress genes that encode cognate GAS elements in their promoter [4,5].

Experimental autoimmune encephalomyelitis (EAE) is a mouse model of multiple sclerosis (MS), a multifocal inflammatory demyelinating disease of the central nervous system (CNS). MS can exhibit distinct clinical phenotypes depending on whether the lesions are localized to the brain, spinal cord or dispersed across CNS compartments [6,7]. EAE is induced in susceptible mouse strains through active immunization with Myelin Oligodendrocyte Glycoprotein (MOG) in Complete Freund’s Adjuvant (CFA) and studies using the EAE model have revealed that inflammation in the distinct microenvironments of the brain and spinal cord results from the recruitment of distinct lymphocyte populations at these CNS sites. Thus, the distinct clinical phenotypes are manifested clinically as atypical EAE (aEAE) or classical EAE (cEAE). The IFN-γ-producing Th1 lymphocyte subset is implicated in the development of cEAE, characterized by the ascending paralysis and infiltration of inflammatory cells into the thoracolumbar spinal cord. In contrast, atypical EAE (aEAE), characterized by abnormal gait imbalance and brainstem or cerebellar inflammation, is mediated by the IL-17-producing Th17 subset [8,9,10]. Thus, the prevailing view is that IL-17 and IFN-γ play important roles in determining whether the inflammatory lesions would localize in the brain or spinal cord [11].

In the context of developing biologics for the suppression or treatment of EAE or MS, it is of note that STAT1 and STAT3 play critical roles in the development of Th1 and Th17 subsets, respectively, and they also regulate the initiation, duration and intensity of proinflammatory immune responses that mediate EAE [2,3]. However, the therapeutic targeting of the STAT1 or STAT3 signaling pathways presents significant challenges because of their intracellular localization. We recently produced and characterized a miniature STAT-specific nanobody derived from camelids (SBT-100) [12]. In this study, we investigated whether SBT-100 can be used as immunotherapy for aEAE in the brainstem and/or cEAE in the spinal cord, the latter of which causes ascending flaccid paralysis.

## 2. Materials and Methods

### 2.1. Mice and Reagents

Six- to eight-weeks-old female C57BL/6J mice were purchased from Jackson Laboratory (Jackson Laboratory, Bar Harbor, ME, USA). Animals were housed at the NIH/NEI animal facility and maintained under 12 -hour light–dark cycle with unlimited access to water and chow, in addition to provision of nutritional supplements at onset of disease. All animal care and procedures were humane and conformed with the National Institute of Health Animal Care and Use Committee guidelines. The experiments were approved and performed under the NIH/NEI Animal Study Protocol (ASP# NEI-597). Singh Biotechnology (Tampa Bay, FL, USA) owns the proprietary rights and provided the SBT-100 [13].

### 2.2. Experimental Autoimmune Encephalitis (EAE) 

EAE was induced using subcutaneous immunization of C57BL/6J mice with 200 µg myelin oligodendrocyte glycoprotein peptide (MOG_35-55_) (Sigma, ST Louis, MO, USA) in CFA emulsion, containing 4.0 mg/mL of heat killed, pulverized *Mycobacterium tuberculosis* strain H37RA. The mice also received two doses of 0.3 µg *Bordetella pertussis* toxin (Sigma, St. Louis, MO, USA) on day 0 and on day 2 post-immunization using intraperitoneal (i.p.) injection in 100 µL of PBS containing 1.0% normal mouse serum. Starting from day 0 of immunization to day 12 post-EAE induction, mice were treated twice daily with 100 µL PBS (untreated group) or SBT-100 (10 mg/kg body weight in 100 µL PBS). In a previous report we established that administering 10 mg/kg SBT-100 intraperitoneally is effective in suppressing inflammation in the retina [12]. Because the retina is also a CNS tissue, we used this dosing regimen in this study. Disease progression was assessed every 2 days and clinical symptoms of EAE were graded according to Choi et al. [14] and Supplementary Figure S1. For histopathological examination, spinal cord and brain were harvested on day 21 post-immunization and fixed in paraformaldehyde and embedded in paraffin. Sections (5 µm) were stained with hematoxylin and eosin (H&E) or used for immunohistochemistry. 

### 2.3. Adoptive Transfer

EAE was induced in wild-type C57BL/6 mice using active immunization with MOG_35-55_ and treated with PBS or SBT-100 as described above. Mice exhibiting clinical features of EAE were sacrificed, and cells isolated from lymph nodes and spleens were reactivated for 72 h in the presence of MOG_35-55_ (20 µg/mL) at 6 × 10^6^/ml. Cells were washed and resuspended in medium and 5 × 10^7^ cells. EAE was then induced in naive syngeneic mice and by day 9 post-immunization, when EAE is already established, we adoptively transferred the MOG-specific encephalitogenic T cells. To demonstrate that SBT-100 can suppress established EAE, some of the mice were treated with SBT-100 beginning on day 12 after adoptive transfer until day 21 post-adoptive transfer. Mice were monitored daily for EAE disease symptoms and scored as earlier described.

### 2.4. Preparation of Single Cell Suspension of CNS Tissues, Draining Lymph Nodes and Spleen

Mice were euthanized and extensively perfused with PBS before lymphoid and CNS tissues were aseptically excised. CNS infiltrated lymphocytes/mononuclear cells were collected from the brain and spinal cord via gentle dissociation using gentleMACS^TM^ (Miltenyi Biotec, Auburn, CA, USA Cat# 130-093-235), digested with Collagenase (1 mg/mL) and DNase (10 µg/mL) and subjected to Percoll gradient centrifugation (Cat# 17089101, Cytiva, Uppsala, Sweden). Centrifuged cells were resuspended in 30% Percoll and layered on 70% Percoll followed by centrifugation at 2500rpm at RT for 25 min. The cells at the 30% and 70% Percoll interface were collected, washed twice, strained through 40 µm cell strainer, and counted using the Vi-Cell XR cell viability analyzer (Beckman Coulter, Brea, CA, USA). Draining lymph nodes and spleens were dissected, and cells freed through teasing in a 40 µm pore cell strainer. Washed cells were suspended in RPMI 1640 medium, erythrocytes lysed in ACK RBC lysis buffer (Quality Biological, Gaithersburg, MD, USA) and lysis was terminated in 10× volume of the medium. Cells were washed (2×), resuspended in medium and seeded at a concentration of 2 × 10^6^/mL.

### 2.5. Intracellular Cytokine Staining and Flow Cytometry Analysis

For intracellular cytokine detection, cells were re-stimulated for 5 h with PMA (50 ng/mL) and ionomycin (500 ng/mL). GolgiPlug (BD Pharmingen, San Diego, CA, USA) was added in the last 2 h, and intracellular cytokine staining was performed using the BD Biosciences Cytofix/Cytoperm kit as recommended. Dead cells were stained with live/dead Fixable Dead Cell Stain Kits (Invitrogen, Carlsbad, CA, USA). 

FACS analysis was performed on cells stained with fluorescent-labeled monoclonal antibodies specific to intracellular cytokines and transcription factors or corresponding isotype antibodies. Dead cells were excluded, and each tube of cells was color-compensated. Quadrant gates were set using isotype controls with less than 0.5% background. FACS analysis was performed on CytoFLEX Flow Cytometer (Beckman Coulter, Indianapolis, IN, USA). Some samples were analyzed using Cytek Aurora System (Cytek, Bethesda, MD, USA). Data analysis was performed on FlowJo version 10.9.0. 

### 2.6. Immunohistochemistry (IHC)

The spinal cord was fixed using formaldehyde, dehydrated in ethanol, and sent for paraffin embedding and sectioning by HistoServ. Inc or NEI Imaging core facility. The slides were deparaffinized and processed for antigen retrieval in Tris EDTA buffer (pH 9.0) using HIER DECLOAKING CHAMBER™ (Biocare, Pacheco, CA) for 15 min at 110 °C followed by slow cooling. Non-specific reactions were blocked using 10% serum, including 1% bovine serum albumin (BSA), 3% skimmed milk, 0.02% sodium azide, and 0.1% Triton X-100. The primary antibody, Rabbit Anti-Mouse CD4 (Ab183685, Abcam, Boston, MA, USA), was incubated overnight at 4 °C in the blocking buffer. The secondary antibody Goat Anti-Rabbit AF568 (A11036, Invitrogen) was incubated for 1 h at RT. The slides were washed and mounted in the EverBrite TrueBlack® Hardset Mounting Medium with DAPI (Biotium, Fremont, CA, USA). Images were acquired using a Zeiss Axio Observer 7 (Zeiss, Oberkochen, Germany). The images acquired using 10× objectives were stitched together using Zeiss Zen-Blue-Edition software (Zeiss, Oberkochen, Germany), and further processed using Imaris 9.9.0.

### 2.7. Cells and Cell Culture 

Lymph nodes were aseptically excised from C57BL/6J mice, and cells freed via teasing in a 40 µm pore cell strainer. Following washing in RPMI 1640 medium, erythrocytes were lysed using 5 mL of ACK RBC lysis buffer (Quality Biological, Gaithersburg, MD, USA) for 3 min and lysis was stopped by adding 10× volume of the medium. All cells were cultured in complete RPMI 1640 media (supplemented with fetal bovine serum (FBS) to a final concentration of 10% and 1× Penicillin-Streptomycin, 2 mM L-glutamine (Life Technologies, Grand Island, NY, USA), 1× HEPES and 5 µM 2-mercaptoethanol) in a humidified incubator at 37 °C and 5% CO_2_. Cells were seeded at a concentration of 2 × 10^6^/mL.

### 2.8. Cell Proliferation Assays

To assess the proliferation of encephalitogenic T cells, draining lymph node cells were seeded at a concentration of 2 × 10^6^/mL and restimulated with MOG_35-55_-peptide (20 µg/mL) for 24, 48 and 72 h with or without different concentrations of SBT-100 (100 µg/mL, 50 µg/mL, 25 µg/mL or 12.5 µg/mL for some experiments). The cells were pulsed with [^3^H]-thymidine (0.5 μCi/10 μL/well) during the last 12 h of each culture timepoints. Presented data are mean count per minute (CPM) ± SD of responses of 6 replicate cultures.

### 2.9. Molecular Modeling to Identify Binding Interactions between SBT-100 and STATs

To study the molecular interactions between SBT-100 and STATs and to identify the target of SBT-100 on the STATs, we performed structural modeling of SBT-100 bound STATs. Crystal structures deposited in Protein Data Bank for STAT3 (PDB: 6QHD) and STAT1 (PDB: 1BF5) were obtained from PDB database in PDB formats. Molecular structure modeling of the SBT-100 amino acid sequence was performed via fold recognition and ab-initio structure prediction methods using Protein Homology/Analogy Recognition Engine (Phyre v2.0) [15] and validated using AlphaFold, an AI system developed by DeepMind that can predict protein folding to high accuracy using only amino acid sequences [16,17]. The modeled SBT-100 structure was subsequently docked to experimentally solved crystal structures of STATs available from the Protein Data Bank (PBD). The docking tool utilized for identifying the binding interactions was HDOCK, a protein–protein and protein–DNA/RNA hybrid algorithm used for docking pairs of molecules via template-based modeling and ab initio free docking [18,19], and validated with AlphaFold multimer [16,17]. Models of docked SBT-100-STATs complexes were visualized using a *PyMOL* implementation on the NIH Biowulf clusters. *PyMOL* is an open source molecular visualization system available from Schrodinger Inc [20].

### 2.10. Statistics 

Data analysis and graph plots were performed on GraphPad Prism 9, using two-tailed unpaired Student’s *t* test for pairwise comparisons. Two-way ANOVA was performed for each time point of the clinical scores. For multiple comparisons, One-way ANOVA with multiple pairwise *t* tests were performed. Data are representative of at least 2 independent experiments and are shown as mean and SEM, and statistical significance for inferences was based on *p* < 0.05. Asterisks in figures denote *p*-values (* *p* < 0.05, ** *p* < 0.01, *** *p* < 0.001, **** *p* < 0.0001). 

## 3. Results

### 3.1. Characterization of the Miniature SBT-100 Nanobody 

**Figure 1:**
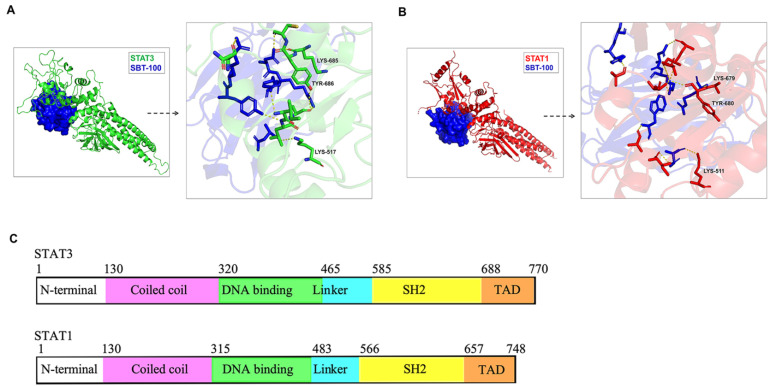
Characterization of SBT-100 nanobody. To define domains of the STAT protein that interact with SBT-100, we used the alphafold2 multimer and HDOCK programs to model the binding of SBT-100 nanobody to STAT proteins. (**A**,**B**) We identified several molecular interactions between SBT-100 and STAT3 (**A**) and STAT1 (**B**). As shown, these are conserved residues in the SH2 domain (Lys-685, Lys-679, Tyr-686, Tyr-680) and the linker domain (Lys-517, Lys-511) of STAT3 and STAT1. (**C**) STAT proteins comprise six domains: N-terminal domain, coiled-coil domain, DNA.

The STAT family of proteins are latent cytoplasmic transcription factors that comprise six domains: the N-terminal domain, coiled-coil domain, DNA-binding domain, linker domain, SH2 domain and Transactivation domain. The binding of cytokines or growth factors to cognate receptors on lymphocytes activate JAKs, resulting in the selective recruitment and activation of requisite STATs that transduce cytokine/growth factor signals to the nucleus. In a recent study, we produced and characterized a miniature STAT-specific nanobody (SBT-100) that suppressed experimental autoimmune uveitis (EAU), an autoimmune disease that serves as a mouse model of human uveitis [12]. Although EAU is mediated via Th17 cells, SBT-100 suppressed Th1 and Th17 cells but had no effects on the Treg subset that requires Foxp3 for its differentiation and development. This finding suggests that SBT-100 might have a global effect on all STAT members. However, in context of the etiology of EAE, STAT1 and STAT3 have been implicated in the EAE/MS and not the other STAT proteins. We therefore examined the interactions between the SBT-100 nanobody and STAT1 or STAT3. The SBT-100 structure was modeled using the amino acid sequence on Phyre and alpha-fold2. Using the alphafold2 multimer and HDOCK programs, we modeled the molecular interactions between STAT1 or STAT3 with the single-domain variable heavy chain (VHH) or SBT-100. For both STAT1 and STAT3, we identified several polar interactions between SBT-100 and conserved residues on the SH2 domain of STAT1 or STAT3 (Figure 1A,B). SBT-100 residues Tyr105, Arg106, Arg112, Arg46, Asn111 and Gln120 form polar interactions with binding partners on SH2 domains of STAT1 and STAT3 as shown (Figure 1A,B; also see the list of binding pairs in Supplementary Figure S2A). Although the SH2 domains of STAT1 and STAT3 are only ~51% identical (Supplementary Figure S2B), we observed interactions involving conserved Tyr686 (STAT3), Tyr680 (STAT1), Lys685 (STAT3), and Lys679 (STAT1) in the SH2 domain and Lys-517 (STAT3), and Lys511 (STAT1) in the linker domain (Figure 1C). Taken together, these findings establish that the main target of SBT-100 is the SH2 domain and this is consistent with our published report that SBT-100 inhibits the STAT3 pathway of uveitogenic Th17 and Th1 cells that mediate uveitis [12]. Thus, these observations are the impetus to investigate whether SBT-100 would be effective in suppressing encephalitogenic Th1 and Th17 cells that mediate inflammation in the brain or spinal cord [11]. 

### 3.2. SBT-100 Ameliorates EAE by Suppressing Inflammation in the Brain and Spinal Cord

**Figure 2:**
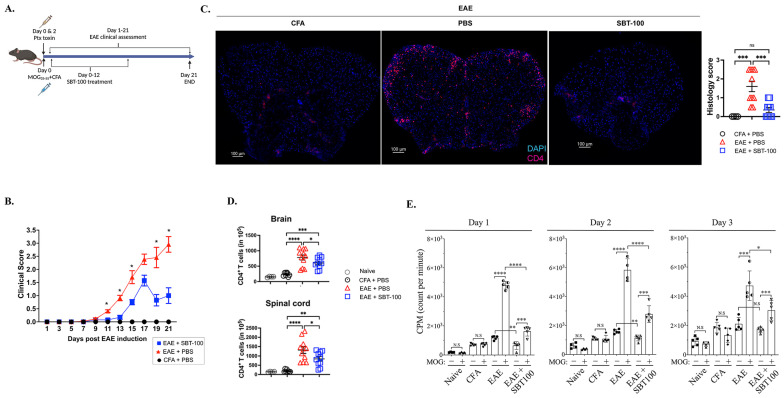
SBT-100 nanobody immunotherapy ameliorates autoimmune encephalomyelitis. (**A**) EAE induction and SBT-100 treatment strategy. (**B**) Disease scores assessed by masked investigators. Representative clinical scores of untreated EAE mice versus SBT-100-treated EAE mice show reduced EAE symptoms in SBT-100-treated mice (blue square), n = 10–12. (**C**) Immunohistochemical showing significant increase in CD4^+^ T cells in the spinal cord of control mice compared to SBT-100-treated mice. Plot shows representative histopathology score of untreated EAE mice versus SBT-100-treated EAE mice. (**D**) Number of CD4^+^ T cells in the spinal cord or brain, n = 10–12. (**E**) [^3^H]-thymidine incorporation assay showing that SBT-100 inhibits proliferation of MOG-specific encephalitogenic T cells, n = 5. Data represent at least 2 independent experiments and presented as mean ± SEM. (* *p* < 0.05; ** *p* < 0.01; *** *p* < 0.001; **** *p* < 0.0001).

EAE was induced via the subcutaneous immunization of C57BL/6J mice with MOG_35-55_ in CFA emulsion, as described (Section 2 Materials and Methods). The treatment group was treated with SBT-100 nanobody from day 0 to day 12 post-EAE induction as indicated (Figure 2A). Control mice treated with PBS developed pathognomonic features of EAE, including infiltration of inflammatory cells into the brain and spinal cord, development of flaccid tail or front/hind limb paralysis and, as the disease progressed, some mice became moribund, while these hallmark features of EAE were attenuated in SBT-100-treated mice as indicated by reduced EAE disease scores (Figure 2B). Compared to SBT-100-treated mice, the immunohistochemical analysis of the day 21 spinal cord reveals the increased infiltration of inflammatory cells into the CNS tissues of the untreated mice, which corresponded to higher histology scores (Figure 2C). On day 21 post-immunization, we isolated and quantified CD4^+^ T cells in the brain or spinal cord and observed significantly reduced numbers of T cells in the brain and spinal cord of SBT-100-treated mice (Figure 2D). T cells isolated on day 21 post-immunization were restimulated in vitro with MOG_35-55_ for 3 days and the effect of SBT-100 on the proliferation of the MOG-specific encephalitogenic T cells was quantified using the lymphocyte proliferation assay. As shown here, at each time point analyzed, we observed a significant decrease in the proliferative capacity of T cells stimulated in medium containing SBT-100 compared to control cells that received PBS (Figure 2E). Interestingly, SBT-100 seems to reduce T cell proliferation without MOG. It is, however, of note that STAT3 plays critical roles in regulating lymphocyte activation and proliferation through its interactions with lymphocyte quiescence factors FoxO1/FoxO3a [21,22], suggesting that SBT-100 may also have generalized effects on lymphocyte proliferation [23]. 

These results suggest that SBT-100 suppresses the expansion of encephalitogenic T cells and that the mitigation of EAE using SBT-100 immunotherapy derived in part from a SBT-100-mediated reduction in encephalitogenic T cells and proinflammatory responses that induce inflammation in the brain and spinal cord [8,9,10].

### 3.3. SBT-100 Mediated Targeting of STATs Is Effective in Suppressing cEAE and aEAE

**Figure 3:**
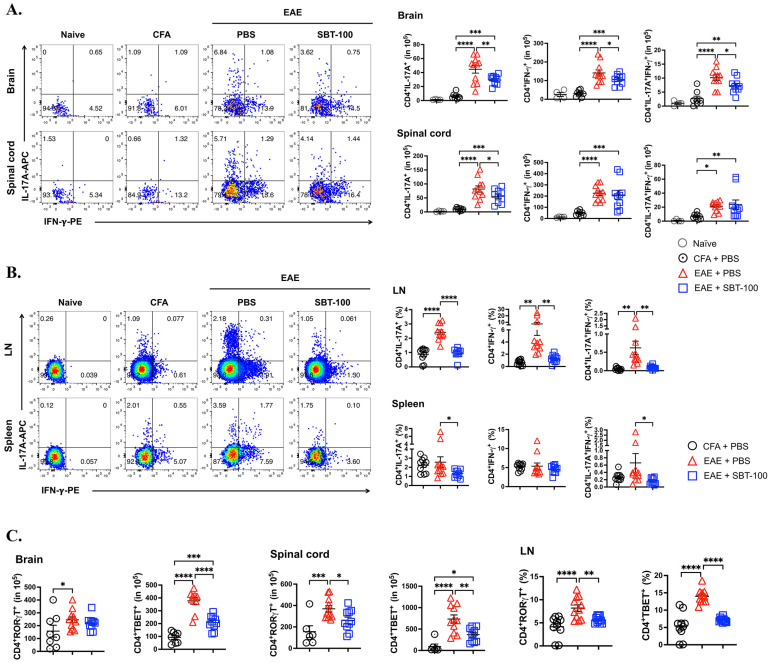
SBT-100 suppress encephalomyelitis by inhibiting pathogenic Th17 and Th1 cells. Lymphocytes isolated from the brain, spinal cord, lymph nodes (LN) or spleen of EAE mice treated with PBS or SBT-100 were subjected to intracellular cytokine staining analysis. (**A**) Flow cytometry plots indicate percentage of T cells in the brain or spinal cord expressing IL-17 and/or IFN-γ. Cytometry percentage bar graphs show suppression of T cells expressing IL-17 and/or IFN-γ in the brain and spinal cord of mice treated with SBT-100. (**B**) Flow cytometry plots indicate percentage of T cells expressing IL-17 and/or IFN-γ in the LN or spleen. Cytometry percentage bar graphs show suppression of T cells expressing IL-17 and/or IFN-γ in the LN or spleen of mice treated with SBT-100. (**C**) Cytometry percentage bar graphs showing expression of ROR-γt or T-bet. Data represent at least 2 independent experiments and presented as mean ± SEM. (* *p* < 0.05; ** *p* < 0.01; *** *p* < 0.001; **** *p* < 0.0001).

IFN-γ-producing Th1 lymphocytes and IL-17-producing Th17 cells play critical roles in the development of EAE in the brain or spinal cord and the commitment to their respective developmental pathway and phenotype requires a sustained activation of STAT1 or STAT3 signal transduction pathway, respectively [24,25]. We therefore examined whether the suppression of EAE in mice treated with SBT-100 derived in part from the inhibition of STAT1 and STAT3 pathways that induce the development of Th1 and Th17 cells or from suppression of effector functions of IFN-γ and IL-17 in the brain or spinal cord. CD4^+^ T cells were isolated from the brain and spinal cord, as well as the spleen and draining lymph nodes, and were analyzed using the intracellular cytokine staining assay. The CD4^+^ T cell representative gating strategy is shown in Figure S3A. The significant infiltration of T cells secreting IL-17A and/or IFN-γ in the brain and spinal cord correlated with the severe EAE in the untreated mice (Figure 3A). Similar analysis shows an increase in T cells secreting IL-17A and/or IFN-γ in the spleen and lymph nodes of these mice (Figure 3B). In contrast, we observed a significant decrease in T cells secreting IL-17A and/or IFN-γ in the SBT-100-treated (Figure 3A,B). The decrease in Th1 and Th17 in these tissues also correlated with the corresponding decrease in the levels of ROR-γt and T-bet which are Th17 and Th1 lineage-specifying transcription factors (Figure 3C and Figure S4). Taken together, these results suggest that SBT-100 mitigates EAE by inhibiting STAT1 and STAT3 pathways required for Th1 and Th17 development and through inducing inflammation in the spinal cord and brain, respectively. 

### 3.4. Lymphocytes from SBT-100-treated EAE Mice Have Reduced Capacity to Transfer EAE

**Figure 4:**
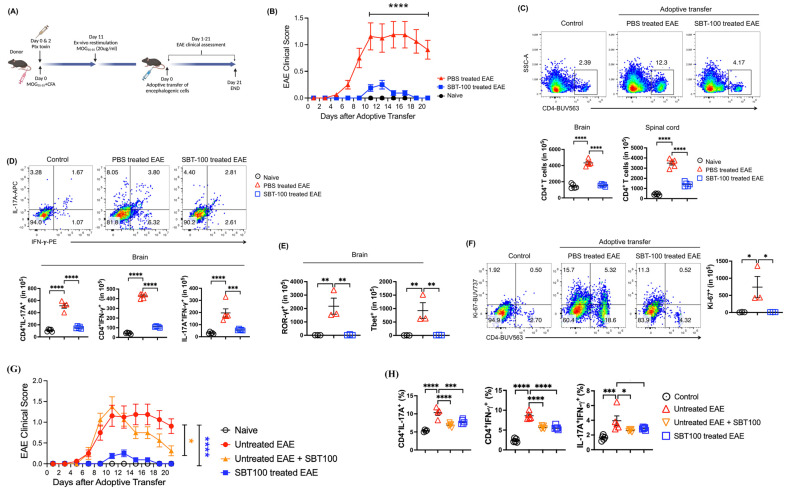
T cells from SBT-100-treated EAE mice have reduced capacity to induce EAE. Cells from the lymph nodes and spleen of control or SBT-100-treated mice with EAE were re-stimulated with MOG_35–55_ for 3 days and the cells (5 × 10^7^ cells/mouse) were transferred to unimmunized naive syngeneic mice. (**A**) Experimental plan for the adoptive transfer EAE experiment. (**B**) EAE clinical score of naive mice, mice that received cells from untreated or SBT-100-treated EAE mice, n = 8. (**C**) Quantification of CD4^+^ T cells in the brain and spinal cord. The representative plot is for brain. (**D**) Intracellular cytokine staining and FACS analysis showing the frequency of IL-17A^+^, IFN-γ^+^ and IL-17A^+^IFN-γ^+^ double positive cells in the brain or spinal cord. (**E**) Frequency of CD4^+^ROR-γt^+^ or CD4^+^T-bet^+^ T cells in the brain or spinal cord. (**F**) Frequency of proliferating T cells in the brain or spinal cord. SBT-100 ameliorates established EAE (**G**). SBT-100 inhibits expansion of Th17 and Th1 (**H**). (* *p* < 0.05; ** *p* < 0.01; *** *p* < 0.001; **** *p* < 0.0001).

Although IL-17 and IFN-γ mediate EAE in the brain and spinal cord, granulocyte macrophage colony-stimulating factor (GM-CSF) knockout mice are resistant to EAE, suggesting that GM-CSF also plays critical role in the development of EAE. GM-CSF is produced by a wide variety of cell types and upregulates the expression of MHC class II and proinflammatory cytokine by microglia, macrophages, and DCs, and promotes the differentiation of CD4^+^ T cells into effector T-cell subsets [26,27,28,29]. To rule out the possibility that the suppression of EAE observed in this study derived from SBT-100 effects on antigen-presenting myeloid cells (APCs), we examined whether the transfer of MOG-specific encephalitogenic CD4^+^ T cells into EAE mice would attenuate the disease. The schematic of the adoptive transfer study is shown (Figure 4A). Briefly, we induced EAE in C57BL/6 mice and isolated MOG-specific encephalitogenic cells from the spleen and draining lymph nodes of EAE mice treated with SBT-100 or untreated EAE mice. We then induced EAE in naive syngeneic mice using active immunization with MOG/CFA and by day 9 post-immunization, when EAE is already established, we adoptively transferred the MOG-specific encephalitogenic T cells. Mice that received cells from the untreated mice developed severe EAE, while mice that receive cells from SBT-100-treated mice developed mild EAE with a delayed onset (Figure 4B). CD4^+^ T cells were isolated from the brain, spinal cord, spleen or lymph nodes of the mice (gating strategy is shown in Figure S3B). The analysis of mice that received encephalitogenic cells from SBT-100-treated mice show a significant correlation of EAE attenuation and decrease in the numbers of CD4^+^ T cells that infiltrated the brain and spinal cord (Figure 4C). We also observed a concomitant reduction in Th17 and Th1 cells secreting IL-17 and/or IFN-γ (Figure 4D) or the transcription factors ROR-γt and T-bet in the brain and spinal cord (Figure 4E). The decrease in inflammatory CD4^+^ T cells also correlated with a significant reduction in the lymphocyte proliferative capacity in mice adoptively transferred with cells from mice treated with SBT-100 (Figure 4F). To directly demonstrate that SBT-100 can suppress established EAE, as would be ideal in clinical settings, we treated a subset of adoptive transfer EAE mice with SBT-100 from day 12 to day 21 post-adoptive transfer. Consistent with disease suppression in the active immunization model, we show that SBT-100 ameliorates established EAE and also inhibited the expansion of Th17 and Th1 (Figure 4G,H). 

## 4. Discussion

Multiple sclerosis is a multifocal inflammatory demyelinating disease of the CNS. In some patients, the disease is restricted to the spinal cord while in others it presents in the brain or other CNS compartments. Although the etiology of MS is not well understood, specific T cell subsets have been identified in histopathological lesions of mice with EAE as well as MS patients, but it is now well established that inflammation is regulated differently in the brain and spinal cord. The secretion of IFN-γ is implicated in the disease, characterized by ascending paralysis while IL-17 secretion mediates the inflammation of the cerebellum, including ataxia [8,9,10]. Consequently, therapeutic strategies that specifically target Th1 responses that mediate spinal cord inflammation may not be effective for suppressing cerebellar ataxia caused by Th17 cells. 

There is currently no cure for MS. However, corticosteroids (oral prednisone and intravenous methylprednisolone) are effective in suppressing relapsing–remitting MS and the therapeutic goal is to promote rapid recovery from attacks or slow down the progression of the disease. However, the side effects of corticosteroids can increase blood pressure, and induce elevated glucose and fluid retention. Besides steroids, treatments that modify the progression of relapsing–remitting MS are also in use and the most prescribed drug among the disease modifying therapies (DMTs) is Interferon beta (IFN-beta). While the side effects of IFN-beta treatment also have adverse effects, including liver damage and reduced drug efficacy due to the development of neutralizing antibodies, the significant adverse effects of these immunosuppressive therapies are the impetus to develop alternative therapies for MS. Importantly, effective therapy for MS must therefore be designed to suppress inflammation in both the spinal cord (mediated by IFN-γ signaling) and the brain (Th17/IL-17), because the specific targeting of T cells in either microenvironment may be ineffective. 

In this study, we have shown that SBT-100, the novel 15 kDa (2.5 nm) nanobody, is effective in suppressing EAE by inhibiting the STAT3 pathway and inflammatory responses mediated by Th17 cells in the brain. This observation is consistent with a recent report showing that SBT-100 suppresses experimental autoimmune uveitis (EAU), a mouse model of human autoimmune uveitis that shares essential immunopathologic features with EAE [12]. Although SBT-100 was initially developed as a STAT3-specific nanobody, the in-depth analysis of the SBT-100 nanobody performed in this study reveals that SBT-100 selectively binds to the Src homology 2 (SH2) domain (approximately 100 amino acids), a highly conserved region required for tyrosine phosphorylation by most STAT proteins [1,30]. Thus, it is not surprising that SBT-100 also suppresses the STAT1 signaling pathway that is required for IFN-γ signaling and Th1 immunological responses in the spinal cord. 

The JAK/STAT plays critical roles in regulating cytokines including IL-6, IL-12, IL-23, IFN-γ, and GM-CSF implicated in MS; consequently, there has been interest in targeting the JAK/STAT pathway as a treatment for MS or EAE. For example, Baricitinib, a JAK 1/2 inhibitor, and AZD1480, another JAK1/2 inhibitor, have been effective in ameliorating EAE [31,32]. On the other hand, Tofacitinib inhibits JAK1, JAK2, JAK3 and is approved by the Food and Drug Administration (FDA) for treating rheumatoid arthritis (RA), psoriatic arthritis and ulcerative colitis [33]. However, it has shown contradictory effects on multiple sclerosis in animal models. Moreover, the use of tofacitinib induced iatrogenic multifocal CNS demyelination in an RA patient [34]. 

## 5. Conclusions

Despite the interest in JAK kinase as a treatment for MS, few studies have focused on the therapeutic use of STAT inhibitors. In this study, SBT-100 suppressed the expansion of Th1 and Th17 in the brain and spinal cord and mice treated with SBT-100 were protected from severe encephalomyelitis. Moreover, the relatively small size of the miniature SBT-100 nanobody facilitates its entry into the CNS, suggesting that SBT-100 immunotherapy can be exploited as a safe therapeutic option for the treatment of neuroinflammatory diseases such as MS. 

## Data Availability

All the data in this paper are presented in Figures and Supplementary figures. Raw data for the same are available upon request to the corresponding author.

## Supplementary Materials

The following supporting information can be downloaded at: https://www.mdpi.com/article/10.3390/cells13121042/s1. **Figure S1.** EAE scoring parameters. **Figure S2. Intermolecular interaction between SBT-100 and STAT. (A)** List of interacting residues and their distance between SBT-100 and the SH2 domain residues of STAT3/STAT1. **(B)** Alignment of the SH2 domains of STAT3 and STAT1. The critical TYR-705 is shown with *. The conserved residues involved in interactions with SBT-100 are shown with #. **Figure S3. Representative gating strategy for CD4+ T cells. (A)** Representative gating strategy for preceding gates for FACS plots shown in Figure 3 and **(B)** Figure 4. **Figure S4. SBT-100 inhibited Th17 and Th1 cells differentiation.** Representative plot of RORγt^+^ and T-bet^+^ T cells. **Figure S5. SBT-100 suppress Th17 and Th1 cells infiltration of the CNS.** Frequency plot of intracellular cytokine staining and FACS analysis showing the number of IL-17A^+^, IFN-γ^+^, IL-17A^+^IFN-γ^+^ double positive cells in spinal cord. Data represent at least 2 independent experiments and presented as mean ± SEM. (*** *p* < 0.001; **** *p* < 0.0001). **Figure S6.** (A) Representative plot of ROR-γt^+^ and T-bet^+^ T cells. The plot for brain is shown as representative. (B) Number of ROR-γt^+^ and T-bet^+^ infiltrating CD4^+^ T cells in the brain and spinal cord.
