# Supplementary material for: A Camelid-Derived STAT-Specific Nanobody Inhibits Neuroinflammation and Ameliorates Experimental Autoimmune Encephalomyelitis (EAE)
Source: Cells. 2024 Jun 16;13(12):1042. doi: 10.3390/cells13121042 (PMC11201538; doi:10.3390/cells13121042)
Supplement: Supplementary file 1 [file cells-13-01042-s001.zip › cells-3018187-supplementary.pdf]

Supplementary Figures

Figure S1

EAE Scoring

0, no clinical symptoms
0.25, 25% of tail flaccid
0.5, 50% of tail flaccid
0.75, 75% of tail flaccid
1.0, limp flaccid tail
then in addition to flaccid tails
1.5, paraparesis of 1 hindlimb
2.0, paraparesis of 2 hindlimb
2.5, paralysis of 1 hindlimb
3.0, paralysis of 2 hindlimb
3.5, paralysis of 2 hindlimb and weakness of 1 forelimb
4.0, paralysis of 2 hindlimb and weakness of 2 forelimb
4.5, complete hindlimbs and forelimbs paralysis
5.0, complete hindlimbs and forelimbs paralysis and reduced responsiveness to external stimulation (sacrifice indicated).

Supplementary Figure S1. EAE scoring parameters.

Figure S2

(A)

List of binding pairs between SBT-100 and STAT3/STAT1 SH2 domains

SH2 DOMAIN		
STAT3 SH2		
SBT-100	STAT3	Å
ASN-111	CYS-687	2.6
ARG-112	TYR-686	1.1
ASN-111	LYS-685	2.5
ARG-112	ALA-555	3
ARG-112	ALA-555	2.9
TYR-113	ILE-553	3.4
TYR-105	LEU-554	3.1
ASN-116	LYS-517	3.5
ARG-106	ASN-624	2.8

STAT1 SH2		
SBT-100	STAT1	Å
ARG-106	THR-704	2.3
TYR-105	ALA-641	2
ARG-46	LYS-679	3.1
ASN-111	SER-682	2.7
ARG-112	TYR-680	2.9
ARG-112	ASP-646	2
ARG-112	ASP-646	3
ARG-112	THR-643	1.8
ARG-112	THR-643	3.1
ARG-112	SER-682	3.3
GLN-120	LYS-511	2
GLN-120	ILE-565	3.4
TRP-118	LYS-567	2.4

(B)

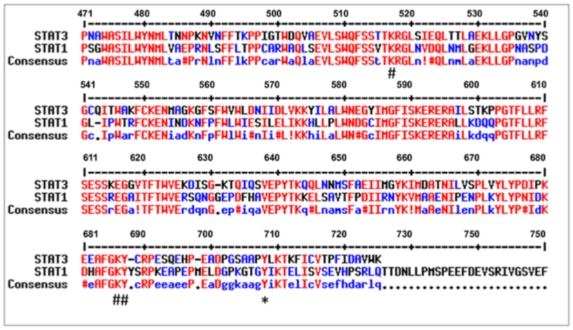

Figure S3

A.

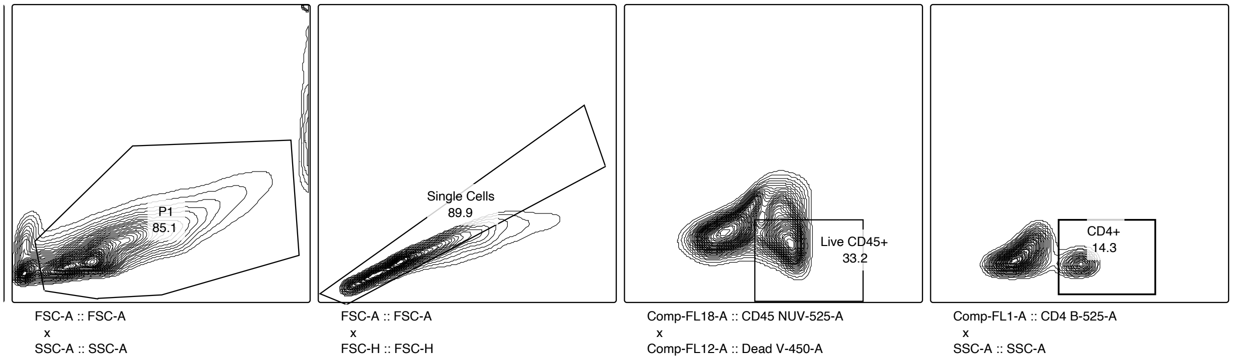

B.

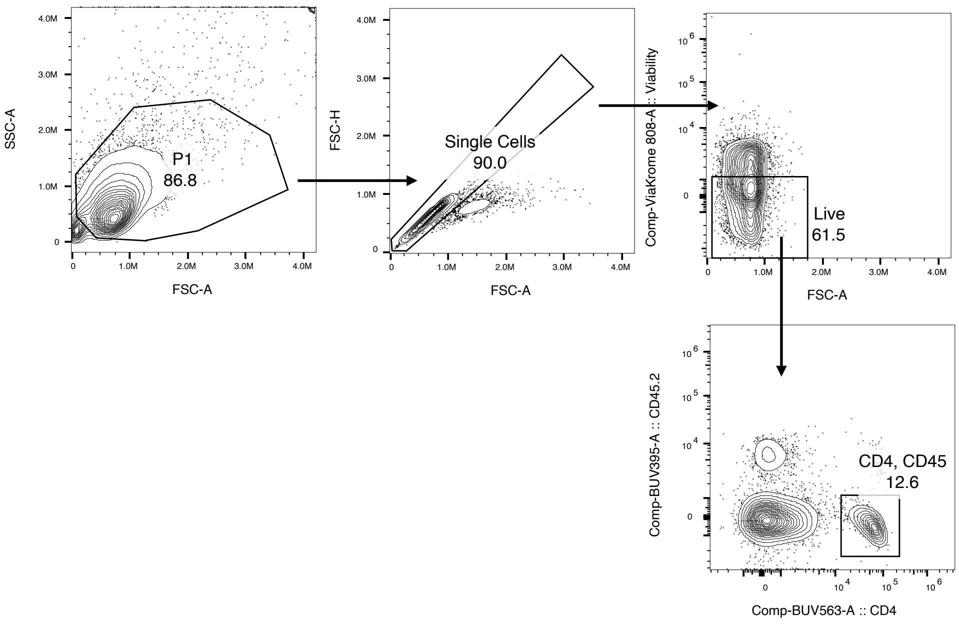

Supplementary Figure S3. Representative gating strategy for CD4+ T cells. (A) Representative gating strategy for preceding gates for FACS plots shown in Figure 3 and **(B)** Figure 4.

Figure S4

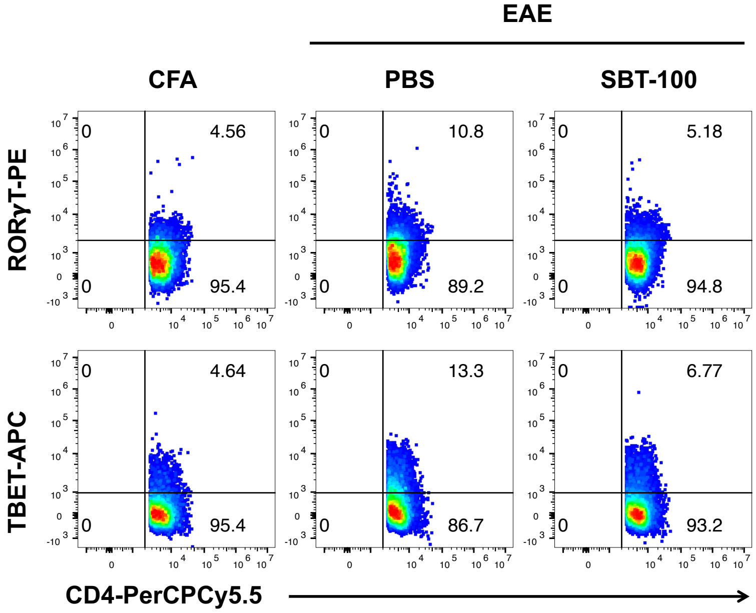

Supplementary Figure S4. SBT-100 inhibited Th17 and Th1 cells differentiation. Representative plot of RORγt$^+$ and Tbet$^+$ T cells.

Figure S5

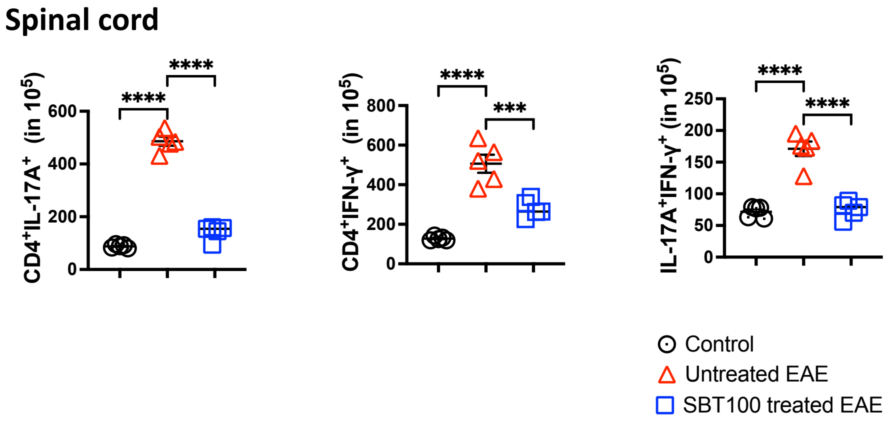

Supplementary Figure S5. SBT-100 suppress Th17 and Th1 cells infiltration of the CNS. Frequency plot of intracellular cytokine staining and FACS analysis showing the number of IL-17A$^+$, IFN-γ$^+$, IL-17A$^+$IFN-γ$^+$ double positive cells in the spinal cord. Data represent at least 2 independent experiments and presented as mean ± SEM. (****p* < 0.001; *****p* < 0.0001).

Figure S6

A

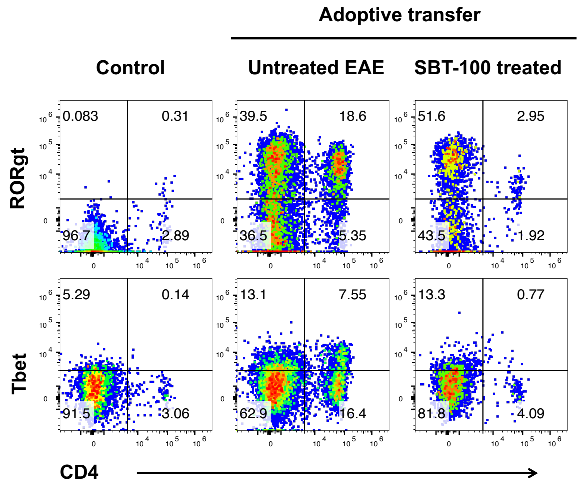

B

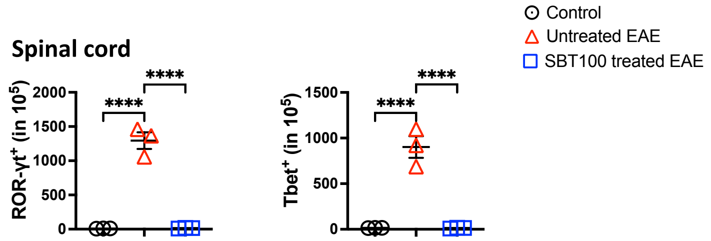

Supplementary Figure S6. (A) Representative plot of RORγt^{+} and Tbet^{+} T cells. The plot for brain is shown as representative. (B) Number of RORgt^{+} and Tbet^{+} infiltrating CD4^{+} T cells in the brain and spinal cord.
